# SMART recovery for youth: a small, exploratory qualitative study examining the potential of a mutual-aid, peer support addictive behaviour change program for young people

**DOI:** 10.1186/s13722-023-00379-w

**Published:** 2023-05-17

**Authors:** Alistair Lum, Despoina Damianidou, Kylie Bailey, Stephanie Cassel, Katherine Unwin, Alison Beck, Peter J. Kelly, Angela Argent, Frank P. Deane, Sophie Langford, Amanda L. Baker, Kristen McCarter

**Affiliations:** 1 The University of Newcastle, University Drive, Callaghan, NSW 2308 Australia; 2 University of Wollongong and Illawarra Health and Medical Research Institute, Northfields Ave, Wollongong, NSW 2522 Australia; 3 SMART Recovery Australia, 33 Saunders St, Pyrmont, NSW 2009 Australia; 4 Headspace Newcastle, 582 Hunter St, Newcastle West, NSW 2302 Australia

**Keywords:** Youth, Addictive behaviours, Mutual-aid group, CBT, Motivational interviewing, Peer worker

## Abstract

**Background:**

SMART (Self-Management and Recovery Training) Recovery is a mutual-aid program informed by cognitive behaviour therapy and motivational interviewing that provides support for a range of addictive behaviours. SMART Recovery has not been adapted to target young people with addictive behaviours despite the potential to overcome important barriers affecting youth engagement in other addiction programs. This study aimed to engage young people and SMART Recovery facilitators in qualitative interviews and focus groups to explore the potential of such a program and gain specific insights for its development.

**Methods:**

We conducted qualitative interviews and a focus group with five young people (aged between 14 and 24 years) and eight key stakeholders (including seven SMART Recovery facilitators) to obtain recommendations on how best to reach, engage, and support young people with addictive behaviours in a tailored SMART Recovery program. Qualitative data was transcribed and analysed using iterative categorization.

**Results:**

Five key themes were identified when developing and delivering youth-targeted SMART Recovery. [1] ‘Discussing personal experiences to promote a shared identity’ refers to the benefits of creating a forum where personal stories are used to connect with others and validate one’s experiences. [2] ‘Flexible and patient approach’ emphasises a preference for facilitators to take a more gentle, less direct approach that allows for discussion beyond addictive behaviours. [3] ‘Balancing information and skills with the space for discussion’ acknowledges that youth want to connect in a variety of ways, beyond discussion of addictive behaviours, and that they wish to lead skill sharing and development. [4] ‘Conveying a community for youth through language’ highlighted the need to focus on connecting youth and to avoid the use of generic language to engage young people. [5] ‘Group logistics and competing demands’ refers to the logistical considerations of implementing a group program for youth that takes into account their competing demands and group accessibility.

**Conclusion:**

The findings point to considerations for developing youth specific mutual-aid groups, in particular a youth-targeted SMART Recovery program, such as by ensuring the conversation is youth-led and with an informal and flexible approach to guide group discussion.

**Supplementary Information:**

The online version contains supplementary material available at 10.1186/s13722-023-00379-w.

Multiple lines of evidence indicate that current youth-targeted addiction-based treatment options may not be meeting the needs of the significant minority of young people with problematic alcohol or other drug (AOD) use, gambling, or other addictive behaviours. First, treatment attendance among adolescents and young adults with AOD problems is low, with rates of young people with AOD disorders attending treatment 4.6 times lower than adults aged over 25 years [1]. Only 9.1% of young Americans with AOD use disorder have engaged with a specialist AOD treatment program [2]. Among youth who attend AOD treatment services, engagement is often poorer with fewer sessions attended before discontinuation in the program [3].

Second, young people report significant barriers contributing to poorer initial and ongoing engagement in substance use and addiction interventions [4]. Young people may believe that they do not have a problem, that engaging with external help is a sign of weakness, and that stopping AOD use would lead to social isolation [5]. Many young people enter treatment under external pressure, such as that from the justice system or families, which can limit treatment engagement [6, 7].

Third, psychotherapeutic programs (e.g., Cognitive Behaviour Therapy [CBT], Multidimensional Family Therapy [MDFT]) for young people with AOD problems that are considered well-established interventions [8] present with specific barriers. For example, family-based interventions rely on caregiver involvement and are more expensive to deliver but no more effective than individual CBT/ Motivational Enhancement Therapy (MET) or non-familial group-based programs in reducing substance use beyond seven months [9, 10]. There is a lack of treatment options for youth targeting gambling behaviours [11], with some evidence supporting CBT-based interventions [12].

Fourth, many programs have been developed or adapted by adults without youth consumer input (e.g., Ozechowski, Becker [13]), a significant flaw that may reduce youth focus and effectiveness [4]. Young people have also expressed that other group members in adult-targeted groups will be older and unrelatable [3, 14].

Despite the range of evidence demonstrating problems in the youth-targeted addiction-based treatments, limited research has focused on resolving them. In one project, Kelly and colleagues developed the integrated Twelve Step Facilitation (iTSF) program for young people using an iterative design that altered the original 12-step program content and structure based on consumer feedback [15]. Important alterations to the program included changing delivery of the content from teaching information and skills to eliciting it through questions and removing the problem-solving content. Kelly and colleagues found no difference between the iTSF program and MET/CBT groups in percentage of days abstinent at post-treatment among 59 youth in a pilot randomised controlled trial [16]. However, iTSF promotes abstinence and incorporates a spiritual or “quasi-religious” component, which may deter youth who view abstinence as unachievable or too restrictive and those who find the religious component incompatible with their own beliefs [3].

SMART Recovery is also a mutual aid program supporting people with addictive behaviours, including those related to alcohol, substances, gambling, shopping, and sex [17]. Each group is led by a trained facilitator using CBT and motivational interviewing (MI) techniques. As a cost-free program that takes a secular, harm-minimisation approach it may offer an alternative group addiction program with fewer attendance barriers. Attendance does not require an addiction diagnosis and is open to people with substance use and other addictive behaviours, such as gambling; an addictive behaviour with limited treatment options and which is often linked to substance use in youth [18]. It is a strengths-based program focused on change rather than problems. Participants are encouraged to attend for as long as they find it helpful, with the goal for engagement to be short-term rather than lifelong. Peer-based support provides the opportunity for group members to share and learn skills based on their own and others personal experiences.

Although SMART Recovery uses evidence-based practices to guide its content and delivery (e.g., CBT, MI, mutual aid), there has been limited empirical evaluation of the program. In a review, Beck and colleagues reported mixed findings from 12 studies when comparing SMART Recovery with 12-step facilitation programs [19]. Group cohesion has been linked to SMART Recovery participants use of cognitive restructuring and is a primary aspect of the program that participants appreciate [20, 21]. The peer-informed group aspect of SMART Recovery offers further potential for young people who are in a developmental stage that is highly responsive to peer influence, especially for risk taking behaviours [22, 23]. Despite the limited data, accumulating empirical and practice-based evidence supports SMART Recovery in reducing addictive behaviours and it may offer the possibility of bridging gaps in the current addiction-based treatment options for youth. This study aimed to explore how SMART Recovery could be tailored to young people through interviews with key stakeholders and youth. The findings will be used in the development of a targeted SMART Recovery program for youth.

## Methods

### Study design

This study is reported according to the consolidated criteria for reporting qualitative studies (COREQ): 32-item checklist which can be found in Additional file 1, (COREQ Checklist) [24]. We applied a qualitative study design using focus groups and interviews. This study was approved by the University of Newcastle Human Research Ethics Committee (H-2020-0087).

### Participants

Two groups of participants were recruited: young people and key stakeholders.

Young people were recruited via headspace Newcastle (Australia), a publicly funded youth mental health service, and paid Facebook advertisements that cost AUD$1000. Young people recruited via headspace were eligible if they were aged from 12 to 25 years and were engaged with a clinician at headspace Newcastle or were a member of the headspace youth reference group (YRG; a consumer feedback group). To be recruited online, young people were eligible if they were aged from 18 to 25 years, resided in New South Wales, and had access to a telephone. We did not limit eligibility to youth with addictive behaviour problems due to the anticipation that youth may not identify as having an addictive behaviour.

Key stakeholders were eligible to participate if they were a trained SMART facilitator, a clinician or researcher collaborating with SMART Recovery, a peer worker working with youth, or a psychologist employed at headspace Newcastle.

### Procedures

Both clinicians and the Community Development Officer at headspace Newcastle were asked to invite young people to participate by providing them brief information about the study and a recruitment flyer. Recruitment flyers were also placed on notice boards at headspace Newcastle. We posted paid advertisements on Facebook and asked investigators to share recruitment flyers through their wider professional network. The target sample size for both sets of participants was based largely on pragmatic considerations, including resources, funding and time with a recruitment period of four months [25]. We aimed to recruit a maximum of 20 young people.

We invited key stakeholders through emails (distributed to SMART Recovery facilitator mailing list, headspace Newcastle clinicians, research team professional networks) via email. We aimed to recruit up to 10 key stakeholders.

We obtained informed consent from all participants and parental informed consent from all parents of young people aged 12–17 years (except for 16–17-year-olds who met mature minor criteria and did not require parental consent under Gillick competence).

Participants were asked to take part in a single focus group (young people recruited via headspace only) or a one-on-one interview over the phone or via videoconference. All focus groups and interviews were audio-recorded and transcribed verbatim. Young people received a AU$25 gift card as renumeration for participation. A semi-structured interview guide was developed by the research team that explored barriers and facilitators to attendance at and engagement in an addictive behaviour peer support group and preferred content and style of delivery of information (see Additional file 2, Interview Guides). The interview guide was not pilot tested. Three authors (1 male, 2 females) who were trained psychologists, had completed a PhD, and with experience in qualitative interviewing conducted interviews and focus groups (AL, DD, KM).

### Analysis

Two investigators (AL and DD) each analysed all transcripts according to thematic analysis theory using the Iterative Categorisation method [26].

## Results

### Participant characteristics

We recruited participants between May 5th and September 1st, 2021. Eleven young people completed the consent to contact form via the Facebook link, and 5 youths from the headspace Newcastle YRG completed the consent to contact. Three consenting to contact through Facebook were ineligible, and seven were uncontactable using the contact details provided. One person from the headspace Newcastle YRG declined to consent after expressing interest in participating.

We recruited five young people. Four participated in a focus group via videoconference and one in an individual interview via telephone. Youth participants ranged from 14 to 24 years. Ten key stakeholders expressed interest in participating, with eight key stakeholders consenting to participate. Interviews were via telephone. Seven stakeholders were trained SMART facilitators, including three counsellors, one youth worker, one caseworker, and the SMART Recovery national program manager. They had between four and 25 years of experience in their field (Median – 16 years).

The interviews and focus group ranged from 33–61 min (Median = 49). No participants elected to provide feedback on the findings.

### Theme 1: discussing personal experiences to promote a shared identity

#### Youth-led discussions and shared experiences

Young people recommended that the program be led by youth, for youth. They reported that the lecture style approach commonly used in schools was not conducive to them engaging with the content or voicing personal experiences. One young person stated that “*If a kid just says an adult [is] running their support group, they’re not going to want to engage in it as much, because it’s someone they can’t probably personally relate to, or connect to. So, if you really stress that it’s for youth and by youth, it’d be a really good thing to attract people to it*.” In practice, youth recommended “*emphasizing the discussions because people are sick of a one-sided discussion*”. Youth noted that connecting socially through enjoyable activities was a way to “*take their focus off the stressful things*” and “*create a safe environment, a place that they can actually feel comfortable or people they can feel comfortable being around*”.

Stakeholders agreed that the most effective way to engage youth and have them learn was by asking them to share their personal experiences with each other, rather than teaching information or skills: *“facilitators aren’t there to tell people what to do*”. One stakeholder recommended that “*you need it to be engaging, you need it to be free-flowing. But most of all, you need it to come from them. You can’t be sitting there lecturing them*.”

Stakeholders noted that youth were less likely than adults to share their experiences unless prompted. One stakeholder summarised many stakeholders’ beliefs by stating that “*group cohesion takes care of itself once [participants] realize they have a shared identity*”. Shared identity was reportedly fostered by one member opening up about their experience in a way to which all members could relate. Stakeholders expressed a belief that youth would continue to share their experiences if the group offered support, respect, and acceptance. One stakeholder reported that “*Peer support … and likeness within the groups, so that they have a shared problem. I find that always very, very helpful, that they’re not alone, that their problem often has a name, that the problem is experienced by other people, and that there is no shame in having it*”.

#### Building intrinsic motivation

Stakeholders reported that many youths, especially males, attended their groups for extrinsic reasons, such as conditions ordered by the justice system, school, or their family. They found that these youth were often disengaged in the group due to a belief that they did not need help, that the group would not be helpful, or opposition to extrinsic forces being placed on them. Stakeholders felt that when disengaged youth experienced benefit early in their attendance their intrinsic motivation to attend increased and they were more likely to engage. Some stakeholders believed this was achieved when youth learnt about consequences others had experienced, had their own experiences validated and supported, and connected with others through shared experiences.

### Theme 2: a flexible and patient approach

#### A softer approach

Stakeholders identified that engaging youth meant taking a different approach to engaging adults. One stakeholder stated that “*when I have a lot of young people in the group, I make sure that they check in and check out* [a standard feature of SMART Recovery]”. They reported that this helped youth actively engage, as they can otherwise be reluctant to participate. Stakeholders reported taking an informal approach which gave them the opportunity to actively listen to young group members and that this increased their engagement, as exemplified by one stakeholder who recommended to not “*let the tools and the skills and the program get in the way of just listening to people and connecting [with] them*”. Praise and reinforcement were also mentioned by some stakeholders as an important way to acknowledge young peoples’ efforts and to build their sense of strength and mastery.

Stakeholders commented on the need for greater patience with youth, as they may need more time to feel comfortable “*thinking a little bit deeper as to [their] reasons as to why [they] did those things and how [they] can change it*”. Stakeholders referred to a “*softer approach*” which involved encouragement, praise, and reinforcement to help youth understand their actions and develop change plans. Some stakeholders suggested that a strengths-based approach that recognises “*what they’re good at or what they like doing*” would also help them engage and learn. Normalising and de-stigmatising young people’s experiences was also identified as an important feature of helping young people feel comfortable sharing their experiences.

#### Acknowledging the broader youth experience

Addictive behaviours were identified by young people and stakeholders as present in a wider context of experiences. Young people identified that drug and alcohol use often occurred in the context of friends and romantic relationships, stress, and as an expected part of the developmental period. They were clear that a conversation that covered the broader context in which addictive behaviours were present was needed.

Stakeholders primarily spoke about drug and alcohol as the key subject for groups but acknowledged that social and emotional factors played a significant role in addictive behaviours. They recommended discussing drug and alcohol use in the context of relationships, stress, and emotions. Some stakeholders recognised that discussing personal experiences in depth may not be within the SMART Recovery guidelines, but that “*It’s difficult to cut [personal stories] off because people are sharing and if you cut it off, then you’re almost not acknowledging about what’s happening for them as their truth*”.

### Theme 3: balancing information and skills with the space for discussion

#### Content delivery

Young people and stakeholders recommended activities, such as art, music, or social games, as a great way to engage youth. Stakeholders suggested educational activities, such as wearing beer goggles while practicing simulated driving, were engaging methods to teach youth. Stakeholders also believed that using visual, rather than written, materials were the best way to share information with young people. Some recommended that facilitators should keep information very brief (e.g., limiting information to “*one topic in two minutes with lots of visual support*”), as youth may not maintain attention for long.

#### Sharing skills

Youth spoke about the value of sharing their personal stories and felt that this should be an important component of the group. Young people felt that they wanted to learn skills to share their personal experiences and thoughts through stories, including one youth who desired “*having that skill of being able to communicate your thoughts, and being able to understand other people’s thoughts*”. Youth reported a belief that this would help foster connection with others and facilitate learning.

Stakeholders believed that unhealthy drug and alcohol use, among other addictive behaviours, could be prevented or managed by developing social-emotional skills. For example, one stakeholder stated that *“[Young people] are going to have to have a way of refusing a substance [in a way] that doesn’t alienate them*” due to the peer pressure that is likely to be placed on them. Stakeholders’ recommendations relating to social-emotional skills extended well beyond refusal skills, and included self-care (e.g., healthy eating, sleeping, and physical activity habits), mind-body connection (e.g., yoga, mindfulness), organisational skills (e.g., time management), emotional literacy and regulation skills, and confidence. Stakeholders also recommended that many of the integral skills already taught in SMART Recovery, such as urge management, problem solving, and goal setting, were as appropriate for youth as they are for older adults.

### Theme 4: conveying a community for youth

#### Broad reach

Youth reported that promoting the program through social media, school, and youth-focused organisations would be necessary to raise awareness of this group and increase young peoples’ engagement. Stakeholders believed that SMART Recovery was poorly promoted to young people and agreed with youth that promotion across a variety of mediums would help reach young people who may be unlikely to seek such a support group themselves. Stakeholders found that networking with youth case workers, legal system representatives, and sports clubs increased the number of youths attending their groups.

#### Meaningful language

Young people stated that terms such as ‘mental health’ and ‘wellbeing’ were overused to the point where they held limited value. One youth commented that “*the way mental health and health in general has been discussed is repetitive. It’s just going to be the same videos that I’ve seen. It’s going to be the same information, and the same support services*”. They also noted that youth were unlikely to respond well to phrases such as ‘addiction’ and ‘addictive behaviours’. Stakeholders agreed, stating that many members of all ages did not believe they had an addiction, let alone any problematic behaviour.

Alternative language was deemed necessary by both participant groups to appeal to young people. Young people suggested conceptualising the group with phrases such as “*youth supporting youth*” or “*connect [with] like-minded people*” to attract young people. Stakeholders suggested that groups be defined as a “*learning community*”. Some facilitators noted that youth had concerns the group would enforce an abstinence approach and recommended that the “*harm minimisation*” approach adopted by SMART Recovery be made clear.

### Theme 5: Group logistics and competing demands

#### Logistical matters

Youth recommended that meetings be held once a fortnight, as regular commitments to school, work, friends, and personal needs meant that weekly attendance may be too great of an obligation for many. They suggested weeknights and weekends as the most appropriate times to attend groups. Youth identified getting to groups as a barrier to attending due to limited transportation options. Videoconferencing was raised as a way to overcome this barrier, with youth agreeing that videoconferencing is now an accepted mode of communication. However, they also mentioned the benefits associated with face-to-face communication. Some facilitators had moved to online groups due to social distancing requirements, with one stakeholder reporting that “*It was actually surprisingly easy, and it’s gone really well*”.

## Discussion

In our interviews and focus group with five young people and eight key stakeholders we identified a range of ways that SMART Recovery could target and support young people with a youth specific mutual aid group. Both young people and stakeholders expressed a clear need for such groups to be led by youth, to have opportunities to share their experiences and connect with others, and to extend beyond addiction.

Youth and key stakeholders were clear that discussions should be youth driven, however youth were less clear about what they wanted from facilitators to support their engagement in the program. Past research suggests that youth seek an informal approach from facilitators who are genuine and who validate their emotions and thoughts [4, 27] and that a lecture format is likely to lead to program disengagement [15]. Key stakeholders agreed and extended on this by adding the benefits of praise, reinforcement, normalising and destigmatising to help youth share their experiences. We suggest that facilitators ask fewer questions and instead use praise and reinforcement strategies that encourage group members to engage in a conversation around health behaviour change. Table 1 provides examples of such strategies. The role of the facilitator is less direct and requires greater patience to allow youth to explore their own and others experiences at their own pace and with minimal facilitator guidance in the discussion topics. This less direct facilitator approach is supported by Kelly and colleagues [15], who found that youth were likely to already have the experiences and knowledge required to make informed choices and to support others in their teachings.

**Table 1:** Reinforcement strategies to support youth discuss health behaviour change topics

Reinforcement strategy	Example
Praise	“Thanks for raising that” or “Great question!”
Reinforcement	“I thought that was a great question about X” or “Wow, what a wonderful idea”
Guide	“You asked something about thoughts/feelings/behaviours, and I’m interested to hear more about that. Does anyone else have something to add to that topic?”
Encourage self-reflection post-praise	“That’s a great question. Why do you think that’s important?”
Modelling	Ask questions using CBT and MI principles as per SMART Recovery guidelines
Summarising at end of session	“I really liked all of the questions about the things that might be triggering certain behaviours, sharing of really practical skills, and most of all the encouragement between everyone”
Pre-teaching	Encourage questions about thoughts, feelings, behaviours in the introduction
Limit attention given to questions that is not CBT/MI related, and interrupt/stop questions that are not appropriate	“I hate to interrupt, but I feel that it’s important that we move away from discussion that encourages unhealthy behaviours”
Prompting	“I’m interested in whether anyone else has had a similar experience and how their emotions or thoughts influenced their behaviour”

One of the most significant needs identified by youth was the need for the group to create opportunities for social connections that support the sharing of personal experiences. Connecting with others in a trusting and safe environment is a critical component of SMART Recovery’s mutual-aid approach. For youth, it may be necessary to incorporate additional measures to establish this sense of trust and safety when compared with adults. In practice, additional measures may begin with facilitators creating opportunities for youth to bond with each other, such as through group social activities as recommended in previous research and by participants in the current study [4, 28]. The presence of a peer worker co-facilitator (i.e., youth with lived experience of mental ill-health who is trained to deliver SMART Recovery) who can share their personal experiences may help strengthen group cohesion and trust while also normalising problems relating to AOD, mental health, or addiction. This is anticipated to increase group members’ sense of safety in sharing their own experiences, as they will have witnessed support and non-judgement when another has opened up to the group. Social support and group cohesion have been linked to better outcomes in SMART Recovery and 12-step facilitation programs [21, 29]. Based on our findings and that of previous research, establishing a shared identity should be considered a foundational element of a youth-targeted SMART Recovery.

To further enhance the current SMART Recovery approach targeting adults, we suggest flexibility to allow topics of discussion to extend beyond addiction. As pointed out by key stakeholders in this study, addictive behaviours are linked to school, relationships, and mental and physical health. Broadening the discussion to include these youth experiences acknowledges that addictive behaviours occur within a wider context of stressors, skills, and resources. A youth-targeted SMART Recovery may offer young people the opportunity to build an awareness of their personal stressors and develop adaptive skills and resources required to overcome such stressors. Developing adaptive coping mechanisms is likely to lead youth away from using AOD or other addictive behaviours as maladaptive coping strategies.

### Strengths and limitations

Initially we aimed to recruit 20 youth participants. At the end of our recruitment period, we had only recruited five young people. We ended recruitment prior to reaching saturation [30], in part due to the slow recruitment rate. Youth participants were not required to have problems with addictive behaviours and thus youth-related data should be interpreted as youth in general, not youth with addictive behaviours. Demographic data, such as that on gender identity or socioeconomic status, was not collected and cannot be used to aid in the interpretation of the findings. The interview guide was open to researcher biases as it was not pilot tested with potential participants. Focus groups and individual interviews can serve two different purposes and can lead to very different information [31]. The data collected from youth which relied on a combination of these methods was generally consistent but its reliability may be limited.

This study was strengthened by the inclusion of two young lived experience researchers who were engaged in the study design and interpretation of findings, a feature recommended in developing youth-focused program [4]. Our partnership with a local, youth-based interagency service was also a component recognised by youth and service providers as integral to engagement [32].

### Future research

The current findings must be validated in a group of young people with addictive behaviours from a range of biographical backgrounds (e.g., gender identity, ethnicity, age, socioeconomic status). Ongoing research is required to transition the current findings from the theoretical stage to modelling, piloting, and if promising, delivery as a controlled trial. We recommend that future research engages stakeholders from youth organisations, education providers, and the justice system to improve engagement with the development and piloting process.

## Conclusion

Our findings indicate that a mutual-aid peer support group tailored for young people should consider conceptualising the group as a community for young people that promotes shared experiences with a flexible approach that accounts for the many psychosocial factors related to addictive behaviours.

## Supplementary Information

**Additional file 1.** COREQ Checklists.

**Additional file 2.** Interview Guides.

## Data Availability

The datasets generated and analysed during the current study are not publicly available due to conditions surrounding individual participants’ privacy but are available from the corresponding author on reasonable request.

## Abbreviations

AOD: Alcohol and other drugs
CBT: Cognitive behavioural therapy
COREQ: COnsolidated criteria for reporting qualitative research
iTSF: Integrated twelve step facilitation
MDFT: Multidimensional family therapy
MET: Motivational enhancement therapy
MI: Motivational interviewing
PHQ: Patient health questionnaire
SMART: Self-management and recovery training
YRG: Youth reference group
