# Supplementary material for: SMART recovery for youth: a small, exploratory qualitative study examining the potential of a mutual-aid, peer support addictive behaviour change program for young people
Source: Addict Sci Clin Pract. 2023 May 17;18:30. doi: 10.1186/s13722-023-00379-w (PMC10190011; doi:10.1186/s13722-023-00379-w)
Supplement: Supplementary file 2 — Additional file 2. Interview Guides. [file 13722_2023_379_MOESM2_ESM.docx]

**Young people Interview Guide - SMART for young people: A qualitative study**

V1 06/06/2021

**INSTRUCTIONS FOR GROUP FACILIATORS: INTRODUCTION**

**Introduce yourself & thank for attending.**

**Confirm the discussion will be audio recorded**: *So that we can make sure we summarise your opinions and thoughts, this discussion will be audio recorded. This is just a reminder that you can choose what you would like to say, and any information about you will be kept confidential. When we summarise this conversation for the team or in any publications, individuals will not be able to be identified*

**Outline the goal for the interview:** *We would like to know what information young people might like included in a group program for young people who are interested in finding support for issues like drugs and alcohol, mental health or social relationships. We would also like to hear your opinions on ideas for some activities and materials that could be included.* *We would also like your thoughts on when the group should run and for how long.*

**Ask if interviewee has any questions before starting.**

**INSTRUCTIONS FOR INTERVIEWER: STEP 1 – SMART INTRODUCTION**

*We are hoping to develop a program here at headspace for young people that is based on SMART. SMART is a free group program that helps people with any behaviours of concern, so things like problem use of drugs, alcohol, cigarettes, gambling, food, shopping, Internet and others. The groups are guided by trained peer workers (people who have a lived experience with these types of problems and recovery) and professionals. Participants come to help themselves and each other using a range of different tools and techniques. We want to know if you think something like this might work for young people. We understand that what works for adults doesn’t always work for young people, and that what adults want might be different to what young people want. We’re here today to find out what you think the program should be like.*

*We want the group to be a safe and supportive place for young people to work through any problems they are experiencing. We want the group to work together to help everyone work towards their own goals and values. Participants in the groups don’t need to identify as ‘having a drug problem’ or a ‘mental health problem’, they just need to bring to the group something they would like to work on. Other than that, we are really open to hearing your thoughts and opinions and your feedback will be super helpful to us in designing this program.*

**INSTRUCTIONS FOR INTERVIEWER: STEP 2 –QUESTIONS**

**Please note the questions presented below do not need to be asked in this order – it is important to respect the group’s priorities and to encourage natural conversational interaction and rapport. Please highlight questions as they are completed to ensure one is not missed.**

Page Break

**SEMI-STRUCTURED GROUP QUESTIONS**

***Setting & Attendance***

- If you were to attend a group program in your local area, what would be some good days and times for it to run?
- Are there any barriers to attending? (If yes - what are these? What would stop you from coming along?)
- What kinds of things would help you be able to get here to attend the program? What would keep you coming back? E.g. a Facebook group?
- How long do you think the group should run for? E.g. 1 hour?
- How do you think we would be able to get the most people here? Facebook page, ads in the paper, printing flyers
- What kind of things would grab your attention or make people your age want to attend?
- What kinds of words should we use to describe the group and what it’s about? For example, if a poster said wellbeing/connections group, would you be interested in coming along?

***Content***

- What sorts of things do you think people your age would like help with?

Prompt: For example, alcohol and drugs (busting myths), mental health, physical health, sexual health, relationships (could be family, friends), managing school or work stress, quality of life

- Would you prefer each week to be focused on a certain topic or to be able to come to the group and learn about and work on whatever you like each week?

***Information, materials***

- SMART is a group where people come together to help each other to work on their goals according to their values. What’s cool is that we don’t tell you what to do. It’s people like you guys helping each other. Sometimes the facilitators will also give some information, but only where you ask for it and it’s helpful.
- What sorts of skills and information might be useful to learn in the group? E.g. meditation, brain science
- Would you prefer information presented in a printed book that you could keep?
- What kinds of activities might be good to include? For example, trivia, quizzes

***General***

Have you attended any other groups? What was good and not so good about them?

Before we finish, is there anything else that you like to add?

**INSTRUCTIONS FOR INTERVIEWER: STEP 3 – CLOSE**

**Check if interviewee found anything distressing today and offer support as appropriate***.*

**Thank you for attending.**

**Interview Semi-structured Guide - SMART for young people: A qualitative study**

V1 02/02/2020

**INSTRUCTIONS FOR INTERVIEWER: INTRODUCTION**

*Thank you for your interest in the study and for volunteering your time. We are aiming to take no more than 60 minutes of your time today.*

*We’re interested in your perspectives on adapting SMART for young people.*

*As explained on the consent form I’d like to audio record the interview. Is it OK to turn on recording now?*

*I am going to start first by quickly asking some questions about your role and experience.*

**INSTRUCTIONS FOR INTERVIEWER: QUESTIONS**

***General***

- Can you describe your current role and how long you have been working in this area?
- Find out what there qualifications are
- Please tell me about your experience running SMART (or other) groups with young people

***Setting & Attendance***

- What have you found to be barriers for young people to attend SMART groups?
- What have you found that helps to improve attendance for young people?
- Please describe any successful strategies you’ve used to promote your groups to increase numbers of new participants.
- Please describe any strategies or factors that improve group cohesion.
- How do you think a group for young people should be described (e.g. wellbeing/connections group) and promoted (e.g. Facebook page, ads in the paper, printing flyers) to be attractive to young people?

***Content and materials***

- What sorts of things do you think young people would like help with? What sorts of goals do they bring to your group to work on?

Prompt: For example, alcohol and drugs (like busting myths), mental health, physical health, sexual health, relationships (could be family, friends), managing school or work stress, quality of life

- What sorts of skills do you think might be useful for participants to learn in the group?
- Do you have any other suggestions for content or materials for a SMART group for young people?

***Mode of support***

- Peer facilitated assistance is central to SMART for adults. Do you think there is a place for facilitator delivered psychoeducation and learning new skills in a SMART group for young people? If yes, what kinds of information and skills do you think would be appropriate?

***General***

Before we finish, is there anything else that you would like to add?

***Closing the interview***

Thank you for your time and for offering your opinions and thoughts.
